# Kidney Outcomes Following Angiotensin Receptor-Neprilysin Inhibitor vs Angiotensin-Converting Enzyme Inhibitor/Angiotensin Receptor Blocker Therapy for Thrombotic Microangiopathy

**DOI:** 10.1001/jamanetworkopen.2024.32862

**Published:** 2024-09-12

**Authors:** Jianbo Li, Qinghua Liu, Xingji Lian, Shicong Yang, Rong Lian, Wenchuan Li, Jianwen Yu, Fengxian Huang, Wenfang Chen, Feng He, Wei Chen

**Affiliations:** 1 Department of Nephrology, The First Affiliated Hospital, Sun Yat-sen University, NHC Key Laboratory of Clinical Nephrology (Sun Yat-Sen University) and Guangdong Provincial Key Laboratory of Nephrology, Guangzhou, China; 2 Department of Nephrology, Jieyang People’s Hospital, Jieyang, Guangdong, China; 3 Department of Geriatrics, Guangzhou First People’s Hospital, The Second Affiliated Hospital of South China University of Technology, Guangzhou, China; 4 Department of Pathology, The First Affiliated Hospital, Sun Yat-sen University, Guangzhou, China; 5 Department of Nephrology, Guangzhou First People’s Hospital, The Second Affiliated Hospital, School of Medicine, South China University of Technology, Guangzhou, China

## Abstract

**Importance:**

Thrombotic microangiopathy (TMA) on kidney biopsy is a pattern of endothelial injury commonly seen in malignant hypertension (mHTN), but treatment strategies are not well established.

**Objective:**

To evaluate the kidney outcomes of angiotensin receptor-neprilysin inhibitor (ARNI), specifically sacubitril/valsartan, vs angiotensin-converting enzyme inhibitor (ACEI) or angiotensin receptor blocker (ARB) therapy for patients with mHTN-associated TMA.

**Design, Setting, and Participants:**

This single-center cohort study enrolled consecutive patients in China diagnosed with mHTN-associated TMA through kidney biopsy from January 2008 to June 2023. Follow-up was conducted until the conclusion of the study period. Data were analyzed in September 2023.

**Exposures:**

Treatment with sacubitril/valsartan or ACEI/ARBs during hospitalization and after discharge.

**Main Outcomes and Measures:**

The primary outcome was a composite of kidney recovery: a 50% decrease in serum creatinine level, decrease in serum creatinine levels to the reference range, or kidney survival free from dialysis for more than 1 month. The secondary and tertiary outcomes were a 15% increase in the estimated glomerular filtration rate (eGFR) relative to baseline and kidney survival free from dialysis, respectively. Propensity score matching (PSM) and Cox proportional hazards regression analysis were used to evaluate the association between sacubitril/valsartan and ACEI/ARB therapy with kidney recovery outcomes.

**Results:**

Among the 217 patients (mean [SD] age, 35.9 [8.8] years; 188 men [86.6%]) included in the study, 66 (30.4%) received sacubitril/valsartan and 151 (69.6%) received ACEI/ARBs at baseline. Sacubitril/valsartan treatment was associated with shorter time to the primary outcome compared with ACEI/ARB treatment (20 of 63 [31.7%] vs 38 of 117 [32.5%]; adjusted hazard ratio [aHR], 1.85; 95% CI, 1.05-3.23). Sacubitril/valsartan treatment was independently associated with shorter time to a 15% increase in eGFR (15 of 46 [32.6%] vs 46 of 83 [55.4%]; aHR, 2.13; 95% CI, 1.09-4.17) and kidney survival free from dialysis (11 of 23 [47.8%] vs 16 of 57 [28.1%]; aHR, 2.63; 95% CI, 1.15-5.88) compared with ACEI/ARB treatment. These differences remained significant in the PSM comparison.

**Conclusions and Relevance:**

In this cohort study, sacubitril/valsartan treatment was associated with a potential kidney function benefit in patients with mHTN-associated TMA compared with ACEI/ARB treatment. The findings suggested that sacubitril/valsartan could be a superior therapeutic approach for managing this serious condition in terms of kidney recovery.

## Key Points

**Question:**

Are there different kidney outcomes among patients diagnosed with malignant hypertension (mHTN)–associated thrombotic microangiopathy (TMA) who received sacubitril/valsartan compared with angiotensin-converting enzyme inhibitor/angiotensin receptor blocker (ACEI/ARB) therapy?

**Findings:**

This cohort study with 217 consecutive patients with mHTN-associated TMA found that sacubitril/valsartan treatment was associated with a shorter time to achieve a composite of kidney recovery outcomes, including a 50% decrease in serum creatinine level, a decrease in serum creatinine level to the reference range, or kidney survival free from dialysis for more than 1 month compared with ACEI/ARB therapy. Similar outcomes were observed for a 15% increase in estimated glomerular filtration rate and kidney survival free from dialysis alone.

**Meaning:**

These findings suggest that sacubitril/valsartan could be a promising therapeutic approach to improve kidney recovery in patients with mHTN-associated TMA.

## Teaser

This cohort study evaluates whether there is a difference in kidney recovery outcomes following angiotensin receptor-neprilysin inhibitor (ARNI) vs angiotensin-converting enzyme inhibitor/angiotensin receptor blocker (ACEI/ARB) therapy among patients with malignant hypertension–associated thrombotic microangiopathy.

## Introduction

Malignant hypertension (mHTN) remains a life-threatening crisis of severe accelerated hypertension that can rapidly progress to end-stage kidney disease.^1^ Thrombotic microangiopathy (TMA), a pathological lesion triggered by endothelial injury and/or dysfunction, is one of the most classic and severe complications of mHTN, with the reported prevalence ranging from 14% to 46% in mHTN.^2,3^ In TMA, the kidneys present with glomerular intracapillary and/or arteriolar thrombosis, along with fragmented red blood cells within capillary lumens and focal ischemia.^4,5^ It has been suggested that the coexistence of TMA in patients with mHTN increases the risk of kidney failure.^2,3,6^ Prompt evaluation and empirical treatment of mHTN-associated TMA are essential to prevent irreversible organ damage and mortality.

The renin-angiotensin-aldosterone system (RAAS), which is highly activated in mHTN, has been established as a major pathogenetic factor in extreme elevations in blood pressure (BP) and microcirculatory damage in mHTN.^7,8,9^ Due to the limited evidence-based therapeutic guidelines for mHTN treatment, management is mainly based on a consensus from clinical experience. The current clinical experience suggests that RAAS blockers, including angiotensin-converting enzyme inhibitors (ACEIs) or angiotensin receptor blockers (ARBs), should be initiated early for patients with mHTN to ameliorate the widespread organ damage.^10,11^ Importantly, recent attention has focused on the potential benefits of combined angiotensin receptor neprilysin inhibitor (ARNI), ie, sacubitril/valsartan, a novel class of drugs with dual inhibition of endogenous natriuretic peptide degradation and renin-angiotensin system activation.^12,13^ Increasing evidence from large randomized clinical trials (RCTs) has shown that sacubitril/valsartan is superior to ACEI/ARBs in terms of cardiovascular and kidney benefits in patients with heart failure (HF) and chronic kidney disease (CKD).^14,15^

Whether the initiation of sacubitril/valsartan therapy is associated with better kidney outcomes compared with the initiation of ACEI/ARBs among patients with mHTN-associated TMA remains unknown. We conducted a prospective cohort study to evaluate the association between the initiation of sacubitril/valsartan vs ACEI/ARBs on kidney function and survival in patients with mHTN-associated TMA.

## Methods

### Study Population and Cohort

This cohort study consecutively enrolled Chinese patients with mHTN and kidney biopsy–proven TMA at the First Affiliated Hospital of Sun Yat-sen University between January 2008 and June 2023. The eligibility criteria were: (1) aged 18 years or older; (2) diagnosed with mHTN; (3) kidney biopsy–proven TMA; (4) receiving treatment with sacubitril/valsartan or ACEI/ARBs; and (5) without missing important baseline data. All patients provided informed consent to receive sacubitril/valsartan or ACEI/ARB treatment according to their own preferences, only after receiving detailed information on the efficacy and safety of both drug options. Patients were divided into the sacubitril/valsartan and ACEI/ARB groups based on treatment during hospitalization and continued treatment after discharge. Despite the lack of formal guidelines for the treatment of mHTN, our approach followed current expert consensus recommendations.^8^ In our setting, patients with mHTN received early intravenous infusion of antihypertensive agents to achieve the target BP range, with gradual introduction of the appropriate tolerable dose of sacubitril/valsartan or ACEI/ARBs over the first 36 to 72 hours. If ACEI was used, it was discontinued for 36 hours before switching to sacubitril/valsartan. We followed the Strengthening the Reporting of Observational Studies in Epidemiology (STROBE) reporting guidelines.

The diagnosis of mHTN was based on the detection of a marked elevation of systolic BP (SBP) levels of 180 mm Hg or greater and/or diastolic BP (DBP) levels 120 mm Hg or greater, accompanied by grade III or IV hypertensive retinopathy according to the Keith-Wagener-Barker classification and/or evidence of imminent or progressive target organ dysfunction secondary to hypertension.^16,17^ Kidney histopathologic lesions in TMA are typically characterized by platelet thrombi and/or fibrinoid necrosis in 1 of 2 broad forms with considerable overlap: (1) predominant arteriolar and lesser arterial or (2) glomerular involvement.^18^ All patients were screened for secondary causes of TMA, such as autoimmune diseases, infections, pregnancy, and drugs. The diagnosis of mHTN-associated TMA was made when a patient was clinically diagnosed with mHTN and clinicopathologically diagnosed with TMA after excluding the secondary causes of TMA. This study was approved by The First Affiliated Hospital of Sun Yat-sen University institutional review board. All patients provided written informed consent. No financial compensation was provided.

### Data Collection and Kidney Histopathology

Routine blood and urine samples were collected within 24 hours of admission, and all patients underwent chest radiography, kidney ultrasonography, and echocardiography. Baseline clinical and demographic data were collected, including body mass index (BMI; calculated as weight in kilograms divided by height in meters squared), BP measurements, history of HTN, daily urinary protein excretion, and serum creatinine level. Prescription data included statins, sacubitril/valsartan, ACEI/ARBs, and other antihypertensive drugs (including α-blockers, β-blockers, and calcium channel blockers). The estimated glomerular filtration rate (eGFR) was calculated using the Chronic Kidney Disease Epidemiology Collaboration equation.^19^ Baseline eGFR was based on baseline serum creatinine levels. Mean arterial pressure (MAP) was defined as one-third of SBP plus two-thirds of DBP.

Percutaneous kidney biopsy specimens were routinely processed according to standard protocols. Kidney biopsies were routinely examined by light microscopy, electron microscopy, and direct immunofluorescence with fluorescein isothiocyanate–conjugated antibodies specific for human immunoglobin (Ig) G, IgM, IgA, C1q, C3, and κ and λ light chains. All kidney biopsies were required to have a minimum of 6 glomeruli and 3 extraglomerular vessels. The number of glomerular scleroses, the degree of tubular atrophy and interstitial fibrosis, and the presence of intravascular thrombi and onion skin–like arteriolar wall thickening were assessed by the senior pathologists (S.Y. and W.C.).

### Study Outcomes

The primary outcome of this study was recovery of kidney function, which was defined as a decrease of serum creatinine level from baseline by more than 50%, a decrease in serum creatinine level to reference range levels (based on the reference range of <1.3 mg/dL [to convert to micromoles per liter, multiply by 88.4], as established by the hospital), or kidney survival free from hemodialysis or peritoneal dialysis for at least 1 month for dialysis-dependent patients. The secondary outcome was an increase of eGFR by more than 15% relative to baseline. The tertiary outcome of this study was kidney survival free from hemodialysis or peritoneal dialysis for dialysis-dependent patients. Dialysis dependency was defined as the need for dialysis support following a diagnosis of mHTN-associated TMA without kidney recovery, even if discharged. All assessors were blinded to the study hypothesis during the dialysis assessment to minimize any potential bias. All patients were followed up by nephrologists and trained nurses through office visits or telephone interviews, and the last follow-up date was June 30, 2023. Patients would be censored immediately at baseline for the study outcomes and continued for follow-up.

### Statistical Analysis

Continuous variables were presented as means and SDs or medians and IQRs. Categorical variables were presented as frequencies and percentages. The analysis of variance for continuous variables and the χ^2^ test or Fisher exact test for categorical variables were performed to compare group differences between the sacubitril/valsartan and ACEI/ARB treatment groups. To adjust for baseline differences and to minimize potential selection bias, we used propensity score matching (PSM) to adjust for nonrandomized treatment allocation to sacubitril/valsartan and ACEI/ARBs.^20,21^ Individual propensities for treatment were estimated using a multivariable probit regression model with baseline covariates adjustment: serum albumin level, eGFR, uric acid level, α-blockers, and kidney histopathology (fibrous necrosis, onion skin lesions, and tubular atrophy or interstitial fibrosis). Nearest-neighbor matching with a 1:1 ratio and a range of 0.2 logits of the standard difference was applied. The efficacy of PSM was evaluated using standardized mean differences (SMDs), with SMDs of 0.1 or less indicating adequate balance for baseline propensity models.

The time to reach study outcomes was estimated using the Kaplan-Meier model, with survival comparisons between the sacubitril/valsartan and ACEI/ARB treatment groups based on a log-rank test. The reverse Kaplan-Meier method was used to estimate median follow-up time. The crude and adjusted hazard ratios (HRs) with 95% CIs were estimated using univariable and multivariable Cox proportional hazards regression models to evaluate the association of different treatments and study outcomes. Variables that were statistically significant in the univariate analysis or were considered clinically associated with kidney outcomes were adjusted. To address the impact of missing follow-up data, we performed a sensitivity analysis assuming that all patients who were censored (due to missing follow-up serum creatinine values) did not experience recovery of kidney function. All analyses were considered statistically significant with a 2-tailed *P* < .05. All analyses were performed using Stata MP version 17.0 (Stata Corp) and R version 3.4.3 (R Foundation for Statistical Computing).

## Results

### Baseline Demographics and Characteristics

**Figure 1.**
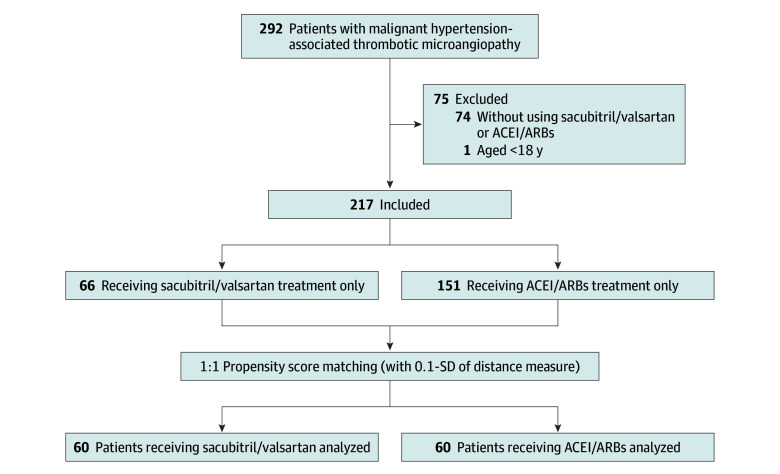
Study Flowchart Abbreviations: ACEI, angiotensin-converting enzyme inhibitor; ARB, angiotensin II receptor blockers.

Of the 217 patients with mHTN-associated TMA (mean [SD] age, 35.9 [8.8] years; 188 men [86.6%]) included, 66 (30.4%) received sacubitril/valsartan treatment, and 151 (69.6%) received ACEI/ARB treatment (Figure 1). The demographic and clinical characteristics of the patients before PSM are reported in Table 1. Compared with patients receiving ACEI/ARB treatment, patients receiving sacubitril/valsartan treatment had lower levels of serum albumin, uric acid, and eGFR. Patients treated with sacubitril/valsartan treatment were more likely to receive α-blocker treatment.

**Table 1.** Baseline Characteristics of Patients, Before and After Propensity Score Matching

Characteristic	Unmatched patients			Propensity score, matched patients		
	Sacubitril/valsartan (n = 66), No. (%)	ACEI/ARBs (n = 151), No. (%)	SMD^a^	Sacubitril/valsartan (n = 60), No. (%)	ACEI/ARBs (n = 60), No. (%)	SMD^a^
Demographic						
Age, mean (SD), y	35.4 (8.5)	36.2 (9.0)	−0.10	35.6 (8.3)	35.6 (10.4)	<0.10
Sex						
Female	6 (9.1)	23 (15.2)	0.19	5 (8.3)	11 (13.3)	<0.10
Male	60 (90.9)	128 (84.8)		55 (91.7)	49 (81.7)	
BMI, mean (SD)	24.9 (3.6)	24.4 (3.8)	0.12	25.0 (3.7)	23.9 (3.6)	<0.10
Smoking	31 (47.0)	69 (45.7)	0.03	29 (48.3)	22 (36.7)	<0.10
Hypertension history	29 (43.9)	67 (44.4)	−0.01	26 (43.3)	23 (38.3)	<0.10
Baseline BP status, mean (SD), mmHg						
SBP	218.3 (26.1)	218.5 (24.5)	−0.01	219.1 (25.5)	219.6 (21.2)	<0.10
DBP	136.7 (21.7)	137.2 (20.0)	−0.02	136.9 (21.6)	136.5 (20.7)	<0.10
MAP	163.9 (20.2)	164.3 (19.0)	0.02	164.3 (19.9)	164.2 (17.9)	<0.10
Laboratory values, mean (SD)						
Hemoglobin, g/dL	10.5 (2.5)	10.9 (2.3)	−0.14	10.8 (2.5)	10.6 (2.3)	<0.10
Serum albumin, g/dL	3.5 (0.5)	3.71 (0.5)	−0.36	3.6 (0.5)	3.6 (0.5)	0.1
Serum sodium, median (IQR), mEq/L	140.0 (138.0-141.0)	139.0 (138.0-141.0)	0.08	140.0 (138.0-141.5)	140.0 (138.0-141.0)	<0.10
Serum potassium, mean (SD), mmol/L	4.2 (0.6)	4.1 (0.6)	0.04	4.2 (0.6)	4.1 (0.5)	<0.10
Total cholesterol, mean (SD), mg/dL	181.5 (54.1)	189.2 (54.1)	−0.12	181.5 (57.9)	189.2 (57.9)	<0.10
Triglycerides, median (IQR), mg/dL	159.3 (115.0-212.4)	159.3 (115.0-212.4)	0.05	141.6 (115.0-194.7)	141.6 (97.4-203.5)	0.1
LDL-C, mg/dL	112.0 (42.5)	119.7 (38.6)	−0.14	108.1 (42.5)	119.7 (42.5)	0.1
Serum creatinine, mg/dL	7.9 (4.0, 9.8)	6.2 (3.2, 9.4)	0.30	7.7 (3.9, 9.8)	6.6 (3.3, 9.7)	<0.10
eGFR, median (IQR), mL/min/1.73 m^2^	8.0 (5.9-18.0)	13.4 (6.6-23.2)	−0.23	8.2 (5.9-18.1)	12.2 (6.6-22.6)	<0.10
Uric acid, mg/dL	1.54 (0.99)	1.83 (0.99)	−0.29	1.62 (1.00)	1.50 (0.95)	<0.10
24-h proteinuria, median (IQR), g/d	1.4 (0.9-2.4)	1.3 (0.7-2.2)	0.06	1.4 (0.9-2.3)	1.5 (0.9-2.3)	<0.10
Abnormal complement C3, No. (%)	5 (7.5)	26 (17.2)	0.18	4 (6.7)	10 (16.7)	<0.10
Ejection fraction, %	61.4 (8.8)	59.7 (9.9)	0.14	60.8 (8.9)	59.8 (9.7)	0.1
ITS, cm	14.1 (2.8)	13.8 (2.5)	0.11	14.2 (2.8)	13.5 (2.6)	<0.10
Base medications, No. (%)						
CCB	62 (93.9)	145 (96.0)	−0.10	56 (93.3)	58 (96.7)	<0.10
β-blocker	55 (83.3)	125 (82.8)	0.02	51 (85.0)	48 (80.0)	<0.10
α-blocker	29 (43.9)	93 (61.6)	−0.17	29 (48.3)	31 (51.7)	<0.10
Sulodexide	34 (51.5)	84 (55.6)	−0.08	31 (51.7)	30 (50.0)	<0.10
Statin	37 (56.1)	74 (49.0)	0.14	34 (56.7)	31 (51.7)	<0.10

Abbreviations: ACEI, angiotensin-converting enzyme inhibitor; ARB, angiotensin II receptor blocker; BMI, body mass index (calculated as weight in kilograms divided by height in meters squared); BP, blood pressure; CCB, calcium channel blocker; DBP, diastolic blood pressure; eGFR, estimated glomerular filtration rate; ITS, interventricular septum thickness; LDL-C, low density lipoprotein cholesterol; MAP, mean arterial pressure; SBP, systolic blood pressure; SMD, standardized mean difference.

SI conversion factors: To convert cholesterol (total and LDL-C) to micromoles per liter, multiply by 0.0259; creatinine to micromoles per liter, multiply by 88.4; hemoglobin and serum albumin to grams per liter, multiply by 10; serum sodium and potassium to micromoles per liter, multiply by 1, triglycerides to micromoles per liter, multiply by 0.0113; uric acid to micromoles per liter, multiply by 0.0595.

^a^ To compare characteristics across different treatment groups, analysis of variance was used for continuous variables and χ^2^ tests were applied for categorical variables.

After PSM, 6 patients treated with sacubitril/valsartan were not matched due to the lack of suitable patients receiving ACEI/ARBs within the specified range, and finally, 60 patients treated with sacubitril/valsartan treatment were matched with 60 patients treated with ACEI/ARB treatment (Figure 1). The baseline characteristics were reassessed after PSM and showed that a good balance had been achieved between the 2 groups (SMD <0.1) (Table 1 and eFigure 1 in Supplement 1).

### Kidney Histopathologic Characteristics

Representative light, electron microscopic, and immunofluorescence findings of mHTN-associated TMA are shown in eFigure 2 in Supplement 1. Light microscopic analysis revealed typical series of pathological changes in TMA, including diffuse winkling of the capillary loop and capsular thickening (eFigure 2A in Supplement 1), marked intimal thickening of renal artery (eFigure 2B in Supplement 1), and vessel walls thickening with so-called onion-peel appearance and luminal occlusion (eFigure 2C in Supplement 1). Electron micrographs of a glomerular capillary loop showed endothelial cell swelling and marked subendothelial widening with flocculent material underneath, leading to capillary luminal narrowing (eFigure 2D in Supplement 1). The results indicated that C3c and C5b-9 staining along the vasculature and/or glomerular capillaries reflected in vivo complement activation in mHTN-associated TMA (eFigures 2E and F in Supplement 1). After PSM, no significant differences were observed between groups for global sclerosis, segmental sclerosis, vascular lesions, onion-skin lesions, tubular atrophy or interstitial fibrosis, and tubular epithelial cell exfoliation (eTable 1 in Supplement 1).

### Sacubitril/Valsartan Treatment vs ACEI/ARB Treatment and Kidney Function Recovery

**Figure 2.**
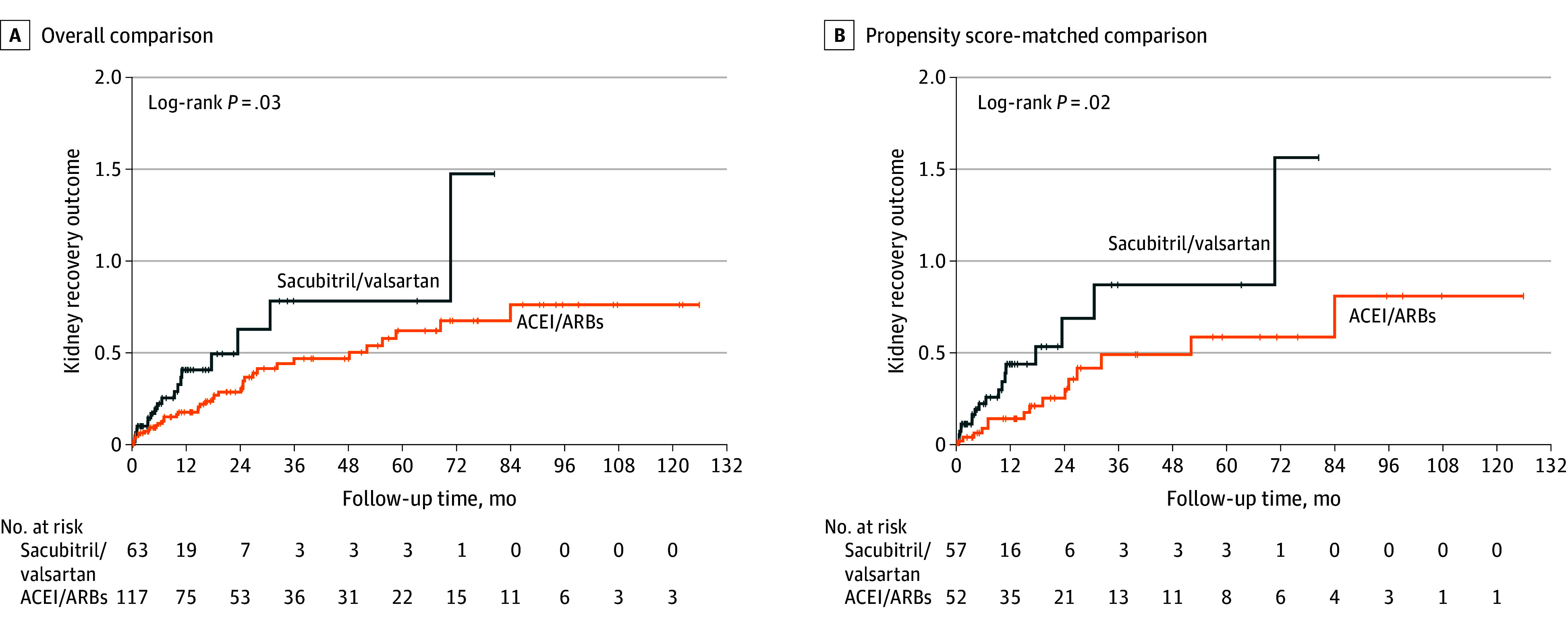
Cumulative Risk of Primary Outcome of Kidney Function Recovery in Patients Receiving Sacubitril/Valsartan or Angiotensin-Converting Enzyme Inhibitor/Angiotensin II Receptor Blocker (ACEI/ARB) Treatment in the Overall Comparison and the Propensity Score–Matched Comparison The primary outcome of this study was defined as a decrease of serum creatinine from baseline by at least 50%, a decrease in serum creatinine to the reference range, or kidney survival free from hemodialysis or peritoneal dialysis for at least 1 month.

With a median follow-up period of 68.5 (95% CI, 35.9-92.1) months, 58 patients (32.2%) had the primary outcome of kidney function recovery. The cumulative probability of sacubitril/valsartan treatment on the hazard of the first occurrence of the primary outcome was significantly higher compared with ACEI/ARB treatment (propensity score–matched comparison, *P* = .02) (Figure 2). In the crude analysis, receiving sacubitril/valsartan treatment was significantly associated with improved primary outcome compared with ACEI/ARB treatment (HR, 1.82; 95% CI, 1.04-3.33; *P* = .04). This difference remained statistically significant after adjustment for both the overall comparison (20 of 63 [31.7%] vs 38 of 117 [32.5%];HR, 1.85; 95% CI, 1.05-3.23; *P* = .04) and the propensity score–matched comparison (HR, 2.22; 95% CI, 1.10-4.55; *P* = .03) (Table 2). A total of 37 cases were censored due to missing follow-up serum creatinine values, including 16 who were lost to follow-up, 11 who died, and 10 who were transferred to other hospital. The sensitivity analysis showed similar results (eFigure 3 in Supplement 1).

**Table 2.** Association Between ACEI/ARBs and Sacubitril/Valsartan Use and Study Outcome Within the Crude Analysis, Multivariable Analysis, and Propensity Score Analyses

Variable	HR (95% CI)	*P* value
**Primary outcome of recovery of kidney function** ^a^		
Patients reaching outcome overall, No. of events/No. of patients at risk (%)	58/180 (32.2)	NA
Sacubitril/valsartan, No. of events/No. of patients at risk (%)	20/63 (31.7)	.92
ACEI/ARB, No. of events/No. of patients at risk (%)	38/117 (32.5)	
Crude analysis^b^	1.82 (1.04-3.33)	.04
Multivariable analysis^c^	1.85 (1.05-3.23)	.04
Propensity score matching^d^	2.22 (1.10-4.55)	.03
**15% increase in eGFR**		
Patients reaching outcome overall, No. of events/No. of patients at risk (%)	61/129 (47.3)	NA
Sacubitril/valsartan, No. of events/No. of patients at risk (%)	15/46 (32.6)	.01
ACEI/ARB, No. of events/No. of patients at risk (%)	46/83 (55.4)	
Crude analysis^b^	1.96 (1.03-3.70)	.04
Multivariable analysis^c^	2.13 (1.09-4.17)	.03
Propensity score matching^d^	2.86 (1.30-6.25)	.009
**Kidney survival free from dialysis**		
Patients reaching outcome overall, No. of events/No. of patients at risk (%)	27/80 (33.8)	NA
Sacubitril/valsartan, No. of events/No. of patients at risk (%)	11/23 (47.8)	.09
ACEI/ARB, No. of events/No. of patients at risk (%)	16/57 (28.1)	
Crude analysis^b^	2.86 (1.28-6.25)	.01
Multivariable analysis^c^	2.63 (1.15-5.88)	.02
Propensity score matching^d^	3.51 (1.18-10.00)	.02

Abbreviations: ACEI, angiotensin-converting enzyme inhibitor; ARB, angiotensin II receptor blocker; eGFR, estimated glomerular filtration rate; HR, hazard ratio; NA, not applicable.

^a^ The primary outcome was defined as a 50% decrease in serum creatinine, a decrease in creatinine to the reference range, or kidney survival free from dialysis therapy for at least 1 month.

^b^ The HRs from the univariable model in all patients from the unmatched study.

^c^ The HRs form the multivariable stratified Cox proportional hazards regression model, with models were adjusted for age, uric acid level, global sclerosis, and tubular atrophy or interstitial fibrosis.

^d^ The HRs from propensity score-matched sample, constructed using 1:1 nearest neighbor matching with calipers of width equal to 0.1 of the SD of the distance measure.

In addition, variables for kidney function recovery in patients with mHTN-associated TMA are shown in eTable 2 in Supplement 1. In the multivariable Cox regression model adjusting for confounders with a *P* < .05 in the univariable regression analysis, sacubitril/valsartan treatment was significantly associated with a shorter time to the primary outcome of kidney function recovery compared with ACEI/ARB treatment (eTable 2 in Supplement 1). The results also showed that a higher proportion of global sclerosis was significantly associated with a lower risk of the primary outcome (adjusted HR, 0.97; 95% CI, 0.95-0.98; *P* < .001) (eTable 2 in Supplement 1).

### Sacubitril/Valsartan Treatment vs ACEI/ARB Treatment and a 15% Increase in eGFR

The time to the first event of a 15% increase in eGFR was shorter in patients receiving sacubitril/valsartan treatment compared with those receiving ACEI/ARB treatment, as shown in eFigure 4 in Supplement 1 (propensity score–matched comparison, *P* = .007). In the crude analysis, sacubitril/valsartan treatment was significantly associated with a 15% increase in eGFR compared with ACEI/ARB treatment (HR, 1.96; 95% CI, 1.03-3.70; *P* = .04). The difference remained statistically significant after adjustment for both the overall comparison (15 of 46 [32.6%] vs 46 of 83 [55.4%]; HR, 2.13; 95% CI, 1.09-4.17; *P* = .03) and the propensity score–matched comparison (HR, 2.86; 95% CI, 1.30–6.25; *P* = .009) (Table 2).

In a univariable regression analysis, statistically significant covariates included treatment with sacubitril/valsartan, age, hypoalbuminemia, eGFR, and global sclerosis (eTable 3 in Supplement 1). After adjusting for these covariates in a multivariable regression analysis, sacubitril/valsartan treatment remained significantly associated with a 15% increase in eGFR compared with ACEI/ARB treatment (eTable 3 in Supplement 1).

### Sacubitril/Valsartan Treatment vs ACEI/ARB Treatment and Dialysis-Free Kidney Survival

Patients receiving sacubitril/valsartan treatment had a significant difference in kidney survival free from dialysis therapy compared with those receiving ACEI/ARB treatment (propensity score–matched comparison, *P* = .02) (eFigure 5 in Supplement 1). In a crude analysis, patients receiving sacubitril/valsartan treatment was significantly associated with improved kidney survival free from dialysis therapy compared with ACEI/ARB treatment (HR, 2.86; 95% CI, 1.28-6.25; *P* = .01). The difference remained statistically significant after adjustment for both the overall comparison (11 of 23 [47.8%] vs 16 of 57 [28.1%]; HR, 2.63; 95% CI, 1.15-5.88; *P* = .02) and the propensity score–matched comparison (HR, 3.51; 95% CI, 1.18-10.00; *P* = .02) (Table 2).

In the multivariable Cox regression model to determine the adjusted significance, sacubitril/valsartan treatment conferred an increased hazard for time to kidney survival free from dialysis compared with ACEI/ARB treatment (Table 3). Additional histologic features significantly associated with a lower risk of kidney survival free from dialysis included the proportion of global sclerosis (adjusted HR, 0.97; 95% CI, 0.96-0.99; *P* = .008), 25% to 50% tubular atrophy or interstitial fibrosis (adjusted HR, 0.05; 95% CI, 0.01-0.21; *P* < .001), and more than 75% tubular atrophy or interstitial fibrosis (adjusted HR, 0.49; 95% CI, 0.25-0.97; *P* = .04) (Table 3).

**Table 3.** Univariable and Multivariable Cox Regression Analysis for Kidney Survival Free From Dialysis Therapy

Variables	Univariable analysis		Multivariable^a^	
	HR (95% CI)	*P* value	Adjusted HR (95% CI)	*P* value
Sacubitril/valsartan vs ACEI/ARB	2.86 (1.28-6.25)	.01	2.63 (1.15-5.88)	.02
Age, per y	1.05 (1.01-1.09)	.04	1.03 (0.98-1.09)	.18
Male sex	0.78 (0.19-3.29)	.74	NA	NA
MAP, mm Hg				
<163	1 [Reference]	NA	NA	NA
≥163	1.28 (0.63-2.63)	.49	NA	NA
Serum albumin level, g/dL				
>3.5	1 [Reference]	NA	NA	NA
≤3.5	1.13 (0.55-2.33)	.75	NA	NA
eGFR, per mL/min/1.73 m^2^	0.98 (0.93-1.02)	.33	NA	NA
Uric acid, mg/dL				
<8.06	1 [Reference]	NA	1 [Reference]	NA
≥8.06	0.36 (0.16-0.78)	.009	0.85 (0.38-1.92)	.71
Complement				
Complement normality	1 [Reference]	NA	NA	NA
Complement abnormality	0.88 (0.33-2.36)	.79	NA	NA
α-Blocker	0.76 (0.36-1.57)	.45	NA	NA
Global sclerosis, %	0.93 (0.87-0.99)	.03	0.97 (0.96-0.99)	.008
Fibrous necrosis	1.19 (0.57-2.48)	.64	NA	NA
Onion dermatoid	0.98 (0.47-2.04)	.96	NA	NA
Tubular atrophy or interstitial fibrosis^b^				
<25%	1 [Reference]	NA	1 [Reference]	NA
25%-50%	0.02 (0.01-0.12)	<.001	0.05 (0.01-0.21)	<.001
50%-75%	1.14 (0.24-5.31)	.87	1.41 (0.31-6.42)	.66
>75%	0.56 (0.23-1.34)	.19	0.49 (0.25-0.97)	.04

Abbreviations: ACEI, angiotensin-converting enzyme inhibitor; ARB, angiotensin II receptor blocker; eGFR, estimated glomerular filtration rate; HR, hazard ratio; MAP, mean arterial pressure; NA, not applicable.

SI conversion factor: To convert albumin to grams per liter, multiply by 10; uric acid to micromoles per liter, multiply by 0.0595.

^a^ Models were adjusted for age, uric acid level, global sclerosis, and tubular atrophy or interstitial fibrosis.

^b^ Trends in degree of tubular atrophy or interstitial fibrosis are presented as 4 degrees based on clinical classification. The *P* for trend for the secondary outcome of kidney survival free from dialysis by biopsy degrees was <.05.

## Discussion

To our knowledge, the present study was the first to evaluate the kidney outcomes of first in-class sacubitril/valsartan vs ACEI/ARB treatment in a large kidney biopsy–proven cohort of patients with mHTN-associated TMA. Our findings indicated that sacubitril/valsartan treatment was associated with better benefits for kidney function recovery, a 15% increase in eGFR, and kidney survival free from dialysis therapy compared with ACEI/ARB treatment in this high-risk population.

The kidney histopathologic features of mHTN are often identical to those seen in certain forms of TMA. The pathophysiology of mHTN-associated TMA involves various interactions related to complement activation, endothelial dysfunction, platelet aggregation, and microvascular thrombosis.^3,17^ Recent studies have highlighted the role of aberrant activation of the kidney alternative complement pathway and pathogenic variants in complement cascade genes as prominent determinants in the severity and kidney outcomes of mHTN-associated TMA or malignant nephrosclerosis.^17,22,23^ Cavero et al^22^ found genetic abnormalities in complement in 19 patients (37%) among those with TMA and mHTN. Timmermans et al^17^ found that complement activation was present in 6 of 9 patients with TMA attributed to severe hypertension. Our immunofluorescence results were consistent with kidney histology staining for complement components in previous studies.^17,22,23^ Endothelial dysfunction is a major contributor to the development of mHTN-associated TMA. Severe and rapidly progressive hypertension damages the endothelial lining of small blood vessels,^24^ leading to the stimulated release of vasoconstrictive substances and RAAS activation.^25^ In response to the cascade of events, increased renin production yields higher levels of angiotensin II, a powerful vasoconstrictor that stimulates endothelin-1 production and promotes inflammation, oxidative stress, and fibrosis.^26^ This rapid activation process creates a self-perpetuating cycle that exacerbates kidney and vascular lesions in other organs. Consequently, pharmacological RAAS inactivation, such as with ACEI/ARB prescription, is a therapeutic approach to limit the specific effects of angiotensin II in mHTN-associated TMA.

Sacubitril/valsartan is a novel therapeutic option that combines the beneficial effects of both angiotensin receptor blockade and inhibition of endogenous natriuretic peptide degradation, resulting in enhanced vasodilation and natriuretic effects. The 2 largest studies, the secondary analysis of the PARADIGM-HF trail and PARAGON-HF trials, demonstrated the kidney benefits of sacubitril/valsartan in patients with HF with reduced ejection fraction compared with ACEI^13,27^ or ARB.^28^ In addition, a meta-analysis of 10 RCTs involving 16 456 patients^29^ showed a kidney protective effect of sacubitril/valsartan in terms of a lower risk of worsening kidney function compared with ACEI/ARBs alone. Similarly, a clinical study in 54 outpatients with HF^30^ showed improved kidney function with sacubitril/valsartan therapy during the 12 months of follow-up. Although the study design is different from our study and most of this evidence is currently limited to patients with HF, the observed benefits of sacubitril/valsartan therapy on kidney function are consistent with our findings. Our study focused on the kidney outcomes of sacubitril/valsartan therapy in mHTN-associated TMA, a unique subgroup of patients with severe hypertension and renal histopathology showing endothelial dysfunction and microvascular thrombosis. Furthermore, our study focused on kidney recovery, due to initially poor kidney function on admission in these patients, which may differ in terms of the outcomes of worsening kidney function or a decrease in eGFR from the previously mentioned studies.

Compared with ACEI/ARBs, the potential pathophysiological mechanisms of sacubitril/valsartan may be explained as follows. First, sacubitril/valsartan inhibits degradation of endogenous natriuretic peptides.^31^ These peptides, such as atrial natriuretic peptide, B-type natriuretic peptide, and C-type natriuretic peptide, play critical roles in promoting natriuresis, diuresis, and vasodilation, while also inhibiting excessive renin and aldosterone release.^32^ By inhibiting neprilysin activity, sacubitril/valsartan increases the levels of natriuretic peptides, thereby enhancing their beneficial cardiovascular and kidney outcomes. Furthermore, the increased bioavailability of renal natriuretic peptides has been shown to improve kidney function and preserve eGFR.^33^ It should be noted that elevated levels of circulating natriuretic peptides may also activate the reflex RAAS and suppress angiotensin II degradation. Therefore, neprilysin inhibition must be combined with RAAS inhibition.^34^ In addition, sacubitril/valsartan blocks the type 1 angiotensin II receptor, which is responsible for adverse cardiovascular and kidney effects, such as vasoconstriction, stimulation of aldosterone release, increased intraglomerular pressure, hypertrophy, fibrosis, and inflammation.^35^ By inhibiting these adverse processes, sacubitril/valsartan can counteract potential long-term damage. In contrast, traditional ACEI/ARBs do not target neprilysin and its beneficial effects on natriuretic peptide action. Moreover, sacubitril/valsartan has been shown to be superior to ACEI/ARBs in reducing the rates of cardiovascular death and hospitalization in patients with HF and reduced ejection fraction.^13,36^ This suggests an overall physiological benefit of sacubitril/valsartan for improved kidney perfusion due to improved cardiac function.^14^

### Limitations

Despite the promising results observed in our study, several potential limitations should be acknowledged. First, we did not report the drug doses in this study due to the different tolerated doses of ACEI/ARB and sacubitril/valsartan for each patient. It was also unclear to us exactly how long after discharge the treatment with sacubitril/valsartan vs ACEI/ARB was discontinued in some patients. Thus, the association of the drug doses and duration with kidney outcomes remains to be further investigated. Second, we recognized that preadmission GFR and albuminuria data were not available for all patients, which are important determinants of outcome. We ensured that baseline GFR and albuminuria were collected within 24 hours of admission and were included in the models to adjust for potential impact. Third, although all TMA diagnoses were identified based on kidney histopathology, we did not have an adequate diagnostic categorization of different TMA etiologies. However, we diagnosed mHTN-associated TMA only after excluding secondary causes of TMA. Fourth, caution should be exercised before applying these findings to patients of other racial and ethnic backgrounds, as the present study was limited to Chinese patients with mHTN-associated TMA. Additionally, the observational nature of the study and potential confounding factors may have influenced the results. Although we used PSM to minimize selection bias, the matched pairs were indeed not independent of each other, and this consideration affected the standard errors and confidence intervals calculated for our outcomes of interest. Thus, unmeasured confounders may still exist.

## Conclusions

In this cohort study, we found that sacubitril/valsartan treatment was associated with favorable kidney function benefits compared with ACEI/ARBs treatment in patients with mHTN-associated TMA. The findings suggested that in terms of kidney recovery, sacubitril/valsartan could be a superior choice for managing this severe condition.

## Notes

Supplement 1. **eFigure 1.** Standardized Mean Difference of Baseline Covariate Adjustment in the Propensity-Score Matched Analysis **eFigure 2.** Representative Light Electron Microscopic and Immunofluorescence Findings of Malignant Hypertension–Associated TMA **eFigure 3.** Cumulative Risk of Primary Outcome of Kidney Function Recovery in Patients Receiving Sacubitril/Valsartan or ACEI/ARBs in the Overall Comparison and Propensity Score–Matched Comparison Based on Sensitivity Analysis **eFigure 4.** Cumulative Risk of a 15% Increase in the eGFR in Patients Receiving Sacubitril/Valsartan or ACEI/ARBs in the Overall Comparison and Propensity Score–Matched Comparison **eFigure 5.** Cumulative Risk of Kidney Survival Free From Dialysis Therapy in Patients Receiving Sacubitril/Valsartan or ACEI/ARBs in Overall Comparison and Propensity Score–Matched Comparison **eTable 1.** Histopathological Findings of Patients Before and After Propensity Score Matching **eTable 2.** Univariable and Multivariable Cox Regression Analysis for Primary Outcome of Recovery of Kidney Function **eTable 3.** Univariable and Multivariable Cox Regression Analysis for a 15% Increase in the eGFR

Supplement 2.
Data Sharing Statement
