# Supplementary material for: Kidney Outcomes Following Angiotensin Receptor-Neprilysin Inhibitor vs Angiotensin-Converting Enzyme Inhibitor/Angiotensin Receptor Blocker Therapy for Thrombotic Microangiopathy
Source: JAMA Netw Open. 2024 Sep 12;7(9):e2432862. doi: 10.1001/jamanetworkopen.2024.32862 (PMC11393719; doi:10.1001/jamanetworkopen.2024.32862)
Supplement: Supplement 1. — eFigure 1. Standardized Mean Difference of Baseline Covariate Adjustment in the Propensity-Score Matched Analysis eFigure 2. Representative Light Electron Microscopic and Immunofluorescence Findings of Malignant Hypertension–Associated TMA eFigure 3. Cumulative Risk of Primary Outcome of Kidney Function Recovery in Patients Receiving Sacubitril/Valsartan or ACEI/ARBs in the Overall Comparison and Propensity Score–Matched Comparison Based on Sensitivity Analysis eFigure 4. Cumulative Risk of a 15% Increase in the eGFR in Patients Receiving Sacubitril/Valsartan or ACEI/ARBs in the Overall Comparison and Propensity Score–Matched Comparison eFigure 5. Cumulative Risk of Kidney Survival Free From Dialysis Therapy in Patients Receiving Sacubitril/Valsartan or ACEI/ARBs in Overall Comparison and Propensity Score–Matched Comparison eTable 1. Histopathological Findings of Patients Before and After Propensity Score Matching eTable 2. Univariable and Multivariable Cox Regression Analysis for Primary Outcome of Recovery of Kidney Function eTable 3. Univariable and Multivariable Cox Regression Analysis for a 15% Increase in the eGFR [file jamanetwopen-e2432862-s001.pdf]

Supplemental Online Content

Li J, Liu Q, Lian X, et al. Kidney outcomes following ARNI vs ACEI/ARB therapy for thrombotic microangiopathy. *JAMA Netw Open.* 2024;7(9):e2432862. doi:10.1001/jamanetworkopen.2024.32862

eFigure 1. Standardized Mean Difference of Baseline Covariate Adjustment in the Propensity-Score Matched Analysis

eFigure 2. Representative Light Electron Microscopic and Immunofluorescence Findings of Malignant Hypertension–Associated TMA

eFigure 3. Cumulative Risk of Primary Outcome of Kidney Function Recovery in Patients Receiving Sacubitril/Valsartan or ACEI/ARBs in the Overall Comparison and Propensity Score–Matched Comparison Based on Sensitivity Analysis

eFigure 4. Cumulative Risk of a 15% Increase in the eGFR in Patients Receiving Sacubitril/Valsartan or ACEI/ARBs in the Overall Comparison and Propensity Score–Matched Comparison

eFigure 5. Cumulative Risk of Kidney Survival Free From Dialysis Therapy in Patients Receiving Sacubitril/Valsartan or ACEI/ARBs in Overall Comparison and Propensity Score–Matched Comparison

eTable 1. Histopathological Findings of Patients Before and After Propensity Score Matching

eTable 2. Univariable and Multivariable Cox Regression Analysis for Primary Outcome of Recovery of Kidney Function

eTable 3. Univariable and Multivariable Cox Regression Analysis for a 15% Increase in the eGFR

This supplemental material has been provided by the authors to give readers additional information about their work.

© 2024 Li J et al. *JAMA Network Open.*

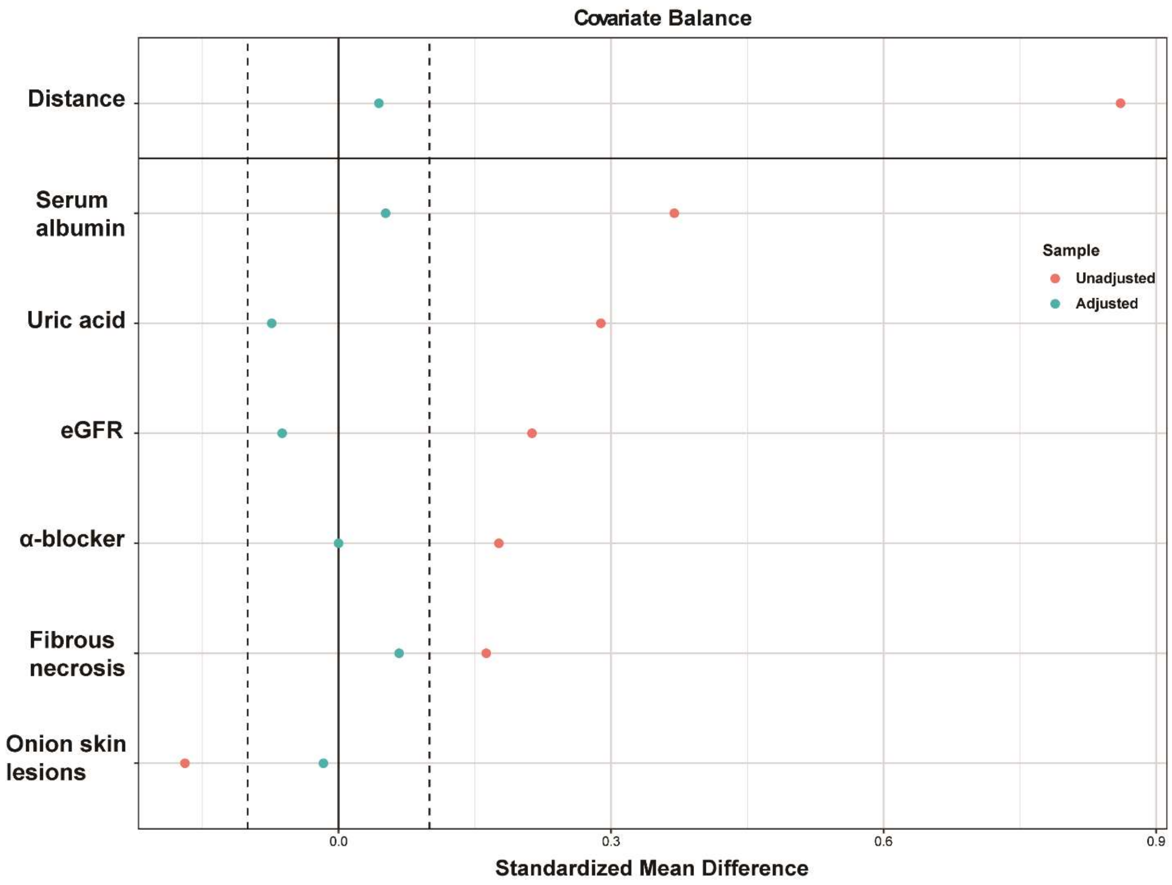

eFigure 1. Standardized Mean Difference of Baseline Covariate Adjustment in the Propensity-Score Matched Analysis

© 2024 Li J et al. *JAMA Network Open.*

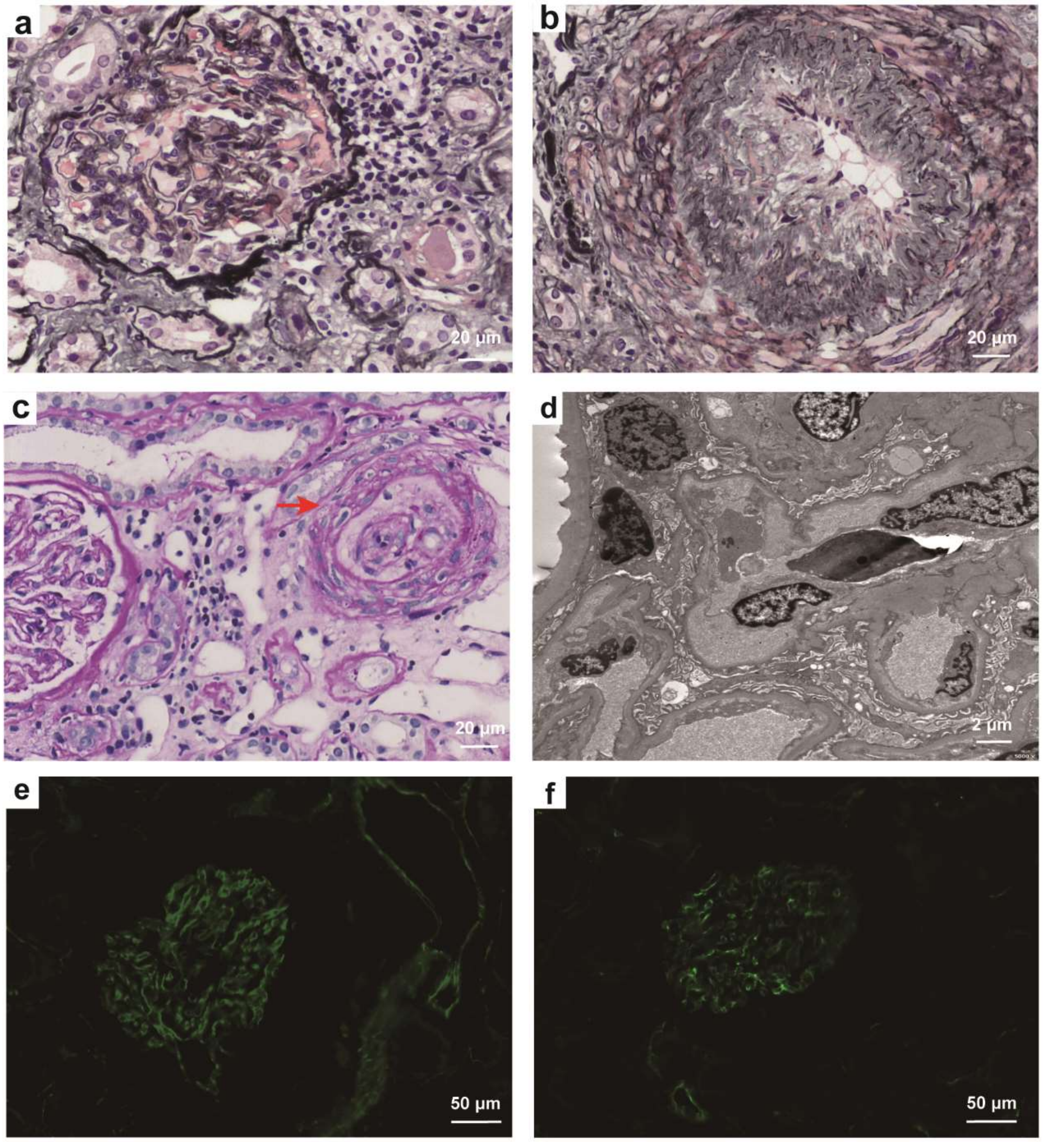

eFigure 2. Representative Light Electron Microscopic and Immunofluorescence Findings of Malignant Hypertension–Associated TMA

(a) Periodic acid-Schiff-Methenamine (PASM) staining showing diffuse winkling of the capillary loop and capsular thickening (original magnification ×400). (b) PASM staining showing marked intimal thickening of renal artery (original magnification ×400). (c) Periodic acid-Schiff (PAS) staining showing vessel walls thickening with "onion-peel" appearance (red arrow) and luminal occlusion (original magnification ×400). Scale bars: 20 µm in a-c. (d) Electron micrograph showing endothelial cell swelling, marked subendothelial widening with flocculent material underneath, leading to capillary luminal narrowing. Scale bar: 2 µm. (e-f) Immunofluorescence images

© 2024 Li J et al. *JAMA Network Open*.

showing C3c (e) and C5b-9 (f) deposition along the arterioles and/or glomerular capillaries. Scale bar: 50 μm.

© 2024 Li J et al. *JAMA Network Open*.

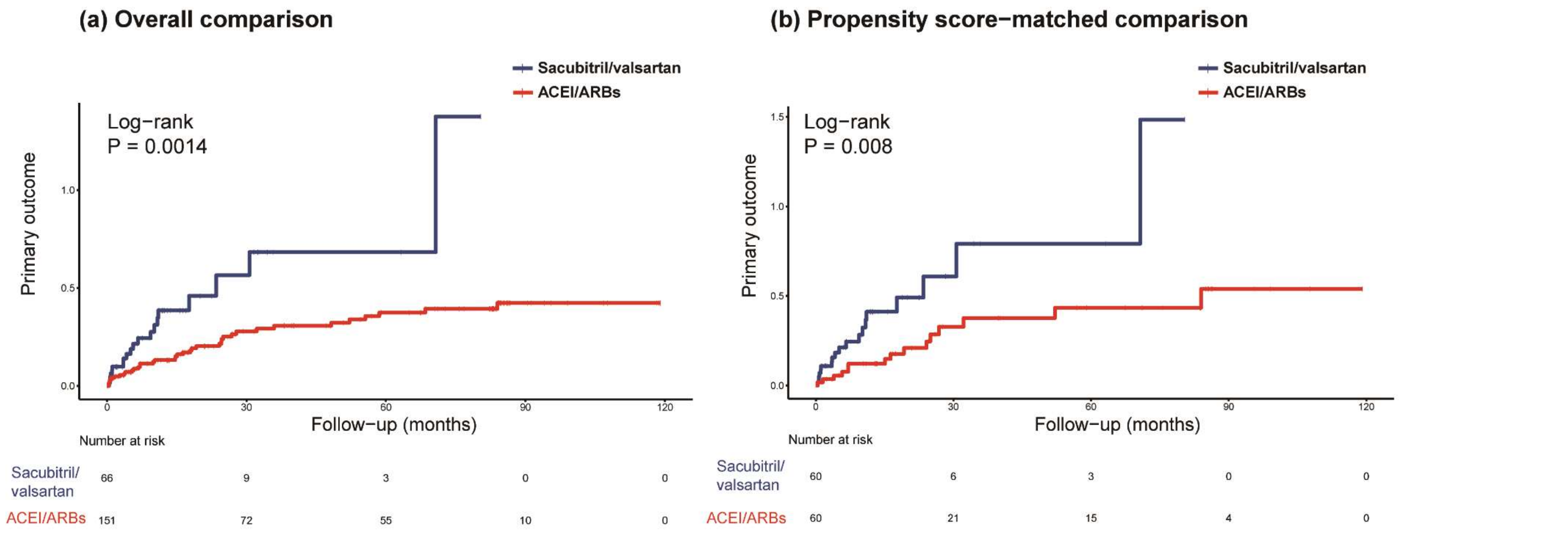

eFigure 3. Cumulative Risk of Primary Outcome of Kidney Function Recovery in Patients Receiving Sacubitril/Valsartan or ACEI/ARBs in the Overall Comparison (a) and Propensity Score–Matched Comparison (b) Based on Sensitivity Analysis

Note: A total of 37 cases were censored due to missing follow-up serum creatinine values.

© 2024 Li J et al. *JAMA Network Open.*

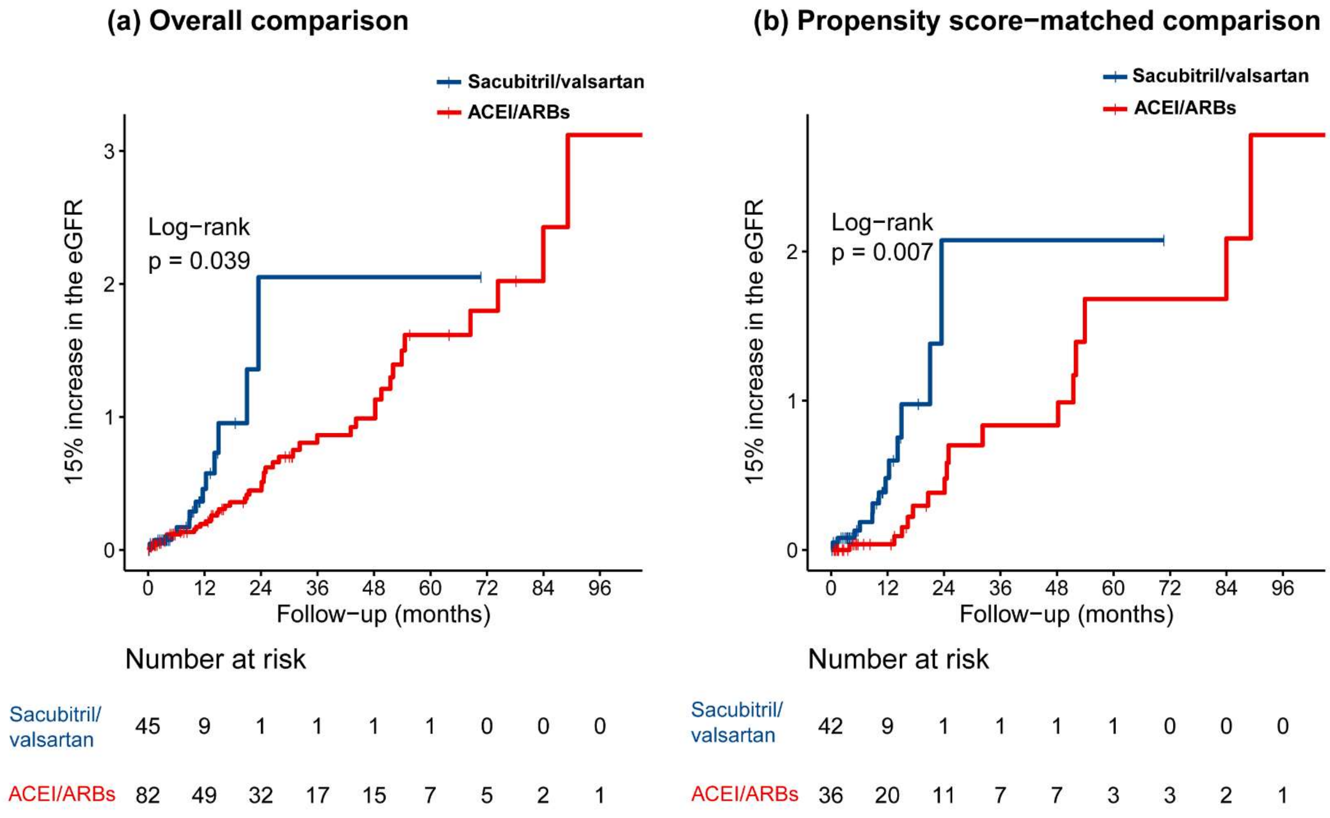

eFigure 4. Cumulative Risk of a 15% Increase in the eGFR in Patients Receiving Sacubitril/Valsartan or ACEI/ARBs in the Overall Comparison (a) and Propensity Score–Matched Comparison (b).

© 2024 Li J et al. *JAMA Network Open.*

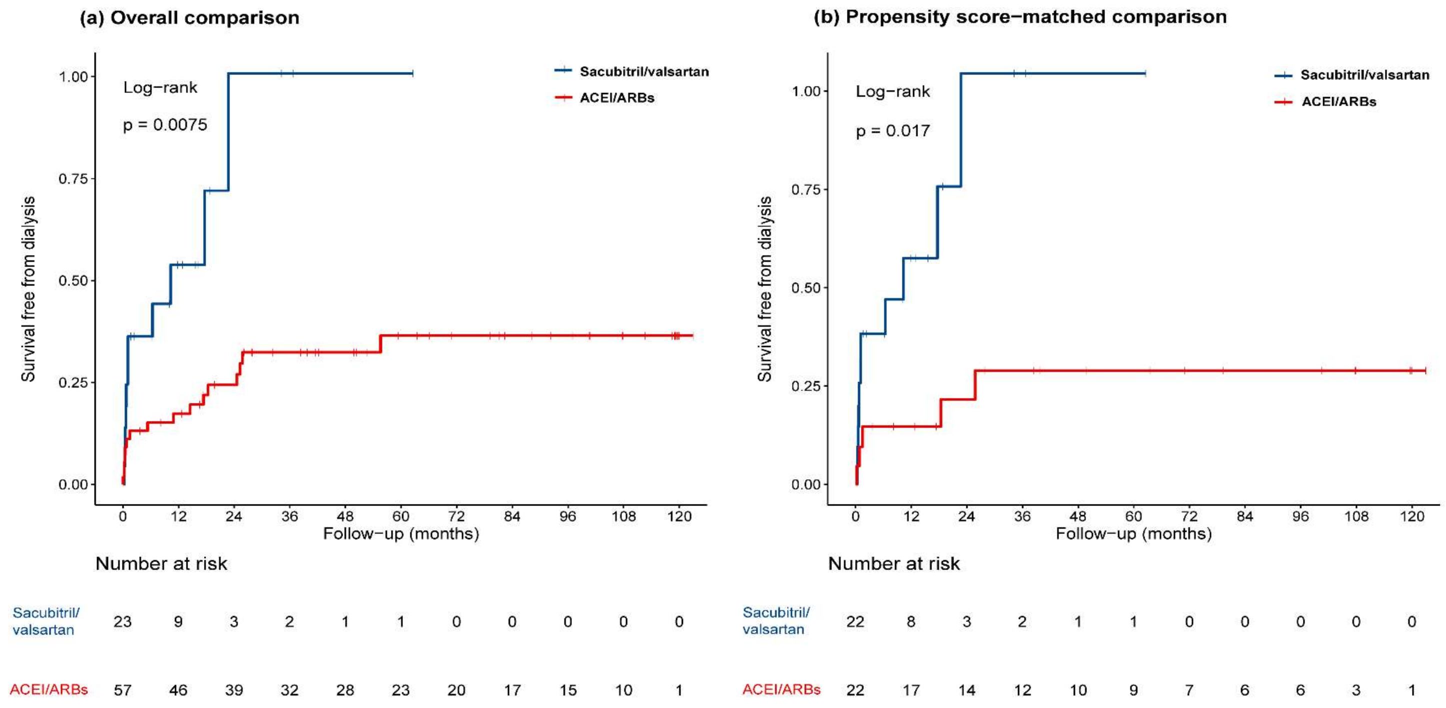

eFigure 5. Cumulative Risk of Kidney Survival Free From Dialysis Therapy in Patients Receiving Sacubitril/Valsartan or ACEI/ARBs in Overall Comparison (n = 80) (a) and Propensity Score–Matched Comparison (n = 44) (b)

Note: The tertiary outcome only included patients who were on dialysis at time zero.

© 2024 Li J et al. *JAMA Network Open.*

eTable 1. Histopathological Findings of Patients Before and After Propensity Score Matching

Biopsy characteristic	Unmatched patients, No. (%)			Propensity score–matched patients, No. (%)		
	Sacubitril/valsartan (n = 66)	ACEI/ARBs (n = 151)	*P*[a]	Sacubitril/valsartan (n = 60)	ACEI/ARBs (n = 60)	*P*[a]
Number of glomeruli, number	26.5 (19.0, 36.0)	23.0 (17.0, 33.0)	0.12	26.0 (18.5, 35.5)	24.5 (17.0, 34.0)	0.67
Percentage of global sclerosis, %	36.6 (16.7, 61.9)	32.4 (20.0, 54.2)	0.94	35.1 (15.5, 54.0)	39.8 (21.4, 60.4)	0.32
Percentage of segmental sclerosis, %	0.0 (0.0, 5.0)	0.0 (0.0, 6.7)	0.23	0.0 (0.0, 5.0)	0.0 (0.0, 4.9)	0.93
**Vascular parameters (arbitrary units), n (%)**						
Hyaline degeneration	37 (56.1)	85 (56.3)	0.97	33 (55.0)	32 (53.3)	0.85
Fibrous necrosis	12 (18.2)	52 (34.4)	0.02	11 (18.3)	13 (21.7)	0.65
Onion-skin lesions	47 (71.2)	82 (54.3)	0.02	41 (68.3)	40 (66.7)	0.85
Intravascular thrombosis	10 (15.2)	21 (13.9)	0.81	10 (16.7)	8 (13.3)	0.61
Intravascular RBC fragments	7 (10.6)	13 (8.6)	0.64	5 (8.3)	6 (10.0)	0.75
**Tubular atrophy/interstitial fibrosis, n (%)**			0.01			0.13
<25%	2 (3.0)	4 (2.6)		2 (3.3)	2 (3.3)	
25–50%	17 (25.8)	30 (19.9)		17 (28.3)	10 (16.7)	
50–75%	29 (43.9)	98 (65.0)		26 (43.3)	39 (65.0)	
>75%	17 (25.8)	16 (10.6)		14 (23.3)	7 (11.7)	
Tubular epithelial cell exfoliation, n (%)	29 (43.9)	48 (31.8)	0.09	26 (43.3)	22 (36.7)	0.46

Abbreviations: ACEI, angiotensin-converting enzyme inhibitor; ARBs, angiotensin II receptor blockers; RBC, red blood cells.

[a] To compare characteristics across different treatment groups, analysis of variance was used for continuous variables and $\chi2$ tests were applied for categorical variables.

© 2024 Li J et al. *JAMA Network Open.*

eTable 2. Univariable and Multivariable Cox Regression Analysis for Primary Outcome of Recovery of Kidney Function[a]

Variables	Univariate analysis		Multivariable analysis[c]	
	**HR (95% CI)**	***P***	**Adjusted HR (95% CI)**	***P***
Sacubitril/valsartan vs. ACEI/ARBs	1.82 (1.04–3.33)	0.04	1.85 (1.05–3.23)	0.04
Age (per, years)	1.01 (0.98–1.03)	0.64	1.01 (0.97–1.02)	0.92
Male gender	0.95 (0.45–2.12)	0.95		
MAP, mmHg				
< 163 mmHg	1(ref)			
≥ 163 mmHg	1.28 (0.80–2.03)	0.31		
Serum albumin, g/dL				
> 3.5 g/dL	1(ref)			
≤ 3.5 g/dL	1.11 (0.67–1.82)	0.69		
eGFR (per mL/min/1.73 m^2)	0.98 (0.96–1.01)	0.17		
Uric acid, mg/dL				
< 8.06 mg/dL	1(ref)			
≥ 8.06 mg/dL	0.89 (0.57–1.42)	0.65		
Complement				
Complement normality	1(ref)			
Complement abnormality	0.62 (0.30–1.27)	0.19		
α-blocker	1.18 (0.73–1.93)	0.52		
Global sclerosis, %	0.96 (0.95–0.98)	< 0.001	0.97 (0.95–0.98)	< 0.001
Fibrous necrosis	1.46 (0.91–2.34)	0.11		
Onion dermatoid	1.16 (0.72–1.89)	0.54		
Tubular atrophy/interstitial fibrosis[b]				
< 25%	1(ref)		1(ref)	
25–50%	0.26 (0.09–0.77)	0.01	0.43 (0.11–1.65)	0.22
50–75%	0.46 (0.20–1.07)	0.07	0.51 (0.18–1.43)	0.20
> 75%	0.98 (0.60–1.61)	0.95	1.09 (0.60–1.96)	0.78

[a] The primary outcome of this study was defined as a decrease of serum creatinine from baseline by > 50%, a decrease in serum creatinine to normal, or renal survival free from hemodialysis or peritoneal dialysis for at least 1 month.

[b] Trends in degree of tubular atrophy/interstitial fibrosis are presented as 4 degrees based on clinical classification. The P for trend for primary outcome by biopsy degrees is < 0.05.

[c] Models were adjusted for age, global sclerosis, and tubular atrophy/interstitial fibrosis.

© 2024 Li J et al. *JAMA Network Open.*

eTable 3. Univariable and Multivariable Cox Regression Analysis for a 15% Increase in the eGFR [a]

Variables	Univariate analysis		Multivariable analysis[b]	
	**HR (95% CI)**	***P***	**Adjusted HR (95% CI)**	***P***
Sacubitril/valsartan vs. ACEI/ARBs	1.96 (1.03–3.70)	0.04	2.13 (1.09–4.17)	0.03
Age (per, years)	0.96 (0.93–0.98)	0.005	0.97 (0.93–0.99)	0.04
Male gender	1.51 (0.79–2.85)	0.22		
MAP, mmHg				
< 163 mmHg	1(ref)			
≥ 163 mmHg	1.53 (0.95–2.46)	0.31		
Serum albumin, g/dL				
> 3.5 g/dL	1(ref)		1(ref)	
≤ 3.5 g/dL	2.08 (1.16–3.74)	0.01	2.26 (1.17–4.34)	0.02
eGFR (per mL/min/1.73 m^2)	1.02 (1.01–1.03)	< 0.001	1.00 (0.99–1.02)	0.15
Uric acid, mg/dL				
< 8.06 mg/dL	1(ref)			
≥ 8.06 mg/dL	1.21 (0.77–1.92)	0.41		
Complement				
Complement normality	1(ref)			
Complement abnormality	0.72 (0.37–1.43)	0.35		
α-blocker	1.14 (0.70–1.85)	0.59		
Global sclerosis, %	1.01 (0.96–1.06)	0.75	0.99 (0.98–1.02)	0.95
Fibrous necrosis	0.91 (0.57–1.44)	0.68		
Onion dermatoid	1.02 (0.64–1.63)	0.93		
Tubular atrophy/interstitial fibrosis[a]				
< 25%	1(ref)			
25–50%	0.34 (0.08–1.45)	0.15		
50–75%	0.52 (0.17–1.56)	0.24		
> 75%	1.10 (0.61–1.97)	0.75		

[a] Trends in degree of tubular atrophy/interstitial fibrosis are presented as 4 degrees based on clinical classification. The P for trend for secondary outcome of 15% increase in the eGFR by biopsy degrees is < 0.05.

[b] Models were adjusted for age, serum albumin, eGFR, and global sclerosis.

© 2024 Li J et al. *JAMA Network Open.*
