# Supplementary material for: Kidney Outcomes Following Angiotensin Receptor-Neprilysin Inhibitor vs Angiotensin-Converting Enzyme Inhibitor/Angiotensin Receptor Blocker Therapy for Thrombotic Microangiopathy
Source: JAMA Netw Open. 2024 Sep 12;7(9):e2432862. doi: 10.1001/jamanetworkopen.2024.32862 (PMC11393719; doi:10.1001/jamanetworkopen.2024.32862)
Supplement: Supplement 2. — Data Sharing Statement [file jamanetwopen-e2432862-s002.pdf]

Data Sharing Statement

Li. Kidney Outcomes Following Angiotensin Receptor-Neprilysin Inhibitor vs Angiotensin-Converting Enzyme Inhibitor/Angiotensin Receptor Blocker Therapy for Thrombotic Microangiopathy. *JAMA Netw Open*. Published September 12, 2024. doi:10.1001/jamanetworkopen.2024.32862

Data
Data available: No

Additional Information
Explanation for why data not available: The datasets used and/or analysed during the current study are available from the corresponding author on reasonable request.
